# Creating novel ornamentals *via* new strategies in the era of genome editing

**DOI:** 10.3389/fpls.2023.1142866

**Published:** 2023-04-14

**Authors:** Chunlian Jin, Liqing Dong, Chang Wei, Muneeb Ahmad Wani, Chunmei Yang, Shenchong Li, Fan Li

**Affiliations:** ^1^ Floriculture Research Institute, Yunnan Academy of Agricultural Sciences, National Engineering Research Center for Ornamental Horticulture, Key Laboratory for Flower Breeding of Yunnan Province, Kunming, China; ^2^ School of Agriculture, Yunnan University, Kunming, China; ^3^ Department of Floriculture and Landscape Architecture, Faculty of Horticulture, Sher-e-Kashmir University of Agricultural Sciences and Technology of Kashmir, Srinagar, India

**Keywords:** ornamental plant, genomics, genetic transformation, new strategy, genome editing

## Abstract

Ornamental breeding has traditionally focused on improving novelty, yield, quality, and resistance to biotic or abiotic stress. However, achieving these goals has often required laborious crossbreeding, while precise breeding techniques have been underutilized. Fortunately, recent advancements in plant genome sequencing and editing technology have opened up exciting new frontiers for revolutionizing ornamental breeding. In this review, we provide an overview of the current state of ornamental transgenic breeding and propose four promising breeding strategies that have already proven successful in crop breeding and could be adapted for ornamental breeding with the help of genome editing. These strategies include recombination manipulation, haploid inducer creation, clonal seed production, and reverse breeding. We also discuss in detail the research progress, application status, and feasibility of each of these tactics.

## 1 Introduction

Flowers not only enhance the aesthetic appeal of their surroundings but also have a positive impact on the psychological well-being of people, thereby making them a product of significant economic value (Wani et al., 2018). Breeders from around the globe have been devoted to developing novel ornamental varieties for millennia, with conventional breeding playing a significant role in this process (Kingsbury, 2009; Bradshaw, 2017). In recent times, the floral industry has witnessed a surge in the availability of a diverse range of cultivars in different ornamental species such as roses, carnations, gerbera, and many more. This has led to the floral industry emerging as one of the most promising businesses across the globe.

While cross-breeding has played an essential role in the development of the modern flower industry, it can be an inefficient process due to the significant time and effort required for emasculation, pollination, and selection. Additionally, genetic variations may emerge at a low frequency, further adding to the inefficiency of the process (Kuligowska et al., 2016). As a result, mutation breeding—using either chemical or radiation mutagenesis—was developed and put into practice to generate genome-wide random mutations, which greatly and efficiently expands genetic variation and diversity. One example of a desirable trait resulting from mutation breeding is the double flower phenotype (Oladosu et al., 2016; Li et al., 2020b). However, due to the random nature of mutation breeding, sifting through a large population of mutagenized plants in hopes of finding the one with the desired trait is both a luxury and an act of extremely good fortune (Mba, 2013). The need for a controllable mutagenesis technology has been urgent in the flower industry. The development of transgenic breeding represents a significant breakthrough, as foreign genes controlling desired traits can be introduced into targeted genomes with precision and control (Raman, 2017). When compared to traditional breeding techniques, this cutting-edge technology opens up new avenues for the generation of additional features with ornamental values, such as the blue-violet-colored flowers seen in roses and carnations (Chandler and Tanaka, 2007). In addition to flower color, it was claimed that other significant commercial traits, including fragrance, longevity, stress tolerance, and disease resistance were improved in different ornamental species (Noman et al., 2017). To date, however, only a few transgenic ornamental cultivars of petunia, rose, and carnation has been bred and employed for commercial purposes due to stringent government regulatory and license requirements and public safety concerns (Bruetschy, 2019; Boutigny et al., 2020).

Genome editing, most notably the CRISPR (clustered regularly interspaced short palindromic repeats) Cas system, has advanced at a remarkable rate in recent years, running in tandem with the advancements in transgenic technology (Ran et al., 2013; Zhang et al., 2014). Considered the next generation of genome engineering, this method offers precise genome editing tools for modifying plant traits (Chen et al., 2019; Wada et al., 2020; Gao, 2021). In addition, the accelerated implementation of genome editing can also be attributed to the rapid progress in sequencing technology (Zhang, 2019; Tang et al., 2020). The major ornamental species including roses (Nakamura et al., 2018; Raymond et al., 2018; Saint-Oyant et al., 2018; Chen et al., 2021), *Rhododendron* (Zhang L. et al., 2017; Soza et al., 2019; Yang et al., 2020; Liu N. et al., 2021; Ma et al., 2021; Shirasawa et al., 2021; Wang X. et al., 2021), orchids (Zhang G. et al., 2017; Chao et al., 2018; Ai et al., 2021), *Chrysanthemum* (Song et al., 2018; Hirakawa et al., 2019), *Helianthus annuus* (Badouin et al., 2017), *Petunia hybrida* (Bombarely et al., 2016), *Platycodon grandifloras* (Kim et al., 2020), *Chimonanthus* (Lv Q. et al., 2020; Shang et al., 2020), and *Paeonia suffruticosa* (Lv S. et al., 2020), have been sequenced and the well-assembled genome data have been released in a very short and intensive period, providing instructive information for understanding the key regulators associated with commercial traits and for the later precise gene editing (Zheng et al., 2021). So far, the CRISPR-based genome editing system has been established in lily (*Lilium pumilum* and *Lilium longiflorum*), orchid (*Phalaenopsis equestris*), *Petunia hybrida*, and *Torenia fournieri* (Kishi-Kaboshi et al., 2018; Ahn et al., 2020). For numerous more ornamental species, the *Agrobacterium*-mediated genetic transformation systems have also been established (Fang et al., 2018; Song et al., 2020). In the foreseeable future, gene editing techniques will play an important role in ornamental plant breeding and make a significant contribution to the enhancement of ornamental features (Bharat et al., 2020; Mishra et al., 2020; Porto et al., 2020).

This article provides a concise overview of the genetic transformation techniques that have been developed for the most important ornamental species. We further raised four breeding strategies based on the published approaches that succeed in either model plant or ornamental crop species and exploit their potential application in ornamental breeding.

## 2 Genetic transformation system: the base of genetic engineering

Both plant molecular biology research and transgenic breeding require efficient and stable plant genetic transformation systems (Morrell et al., 2012). The study of plant genetics has greatly benefited from *Agrobacterium*-mediated plant genetic transformation, the method most typically employed to transfer target gene(s) into plants due to its ease of use, versatility with respect to plasmid size, and modest equipment needs (Hwang et al., 2017). It is also the most used transgenic method for ornamental plants, including herbaceous, woody, bulb, and perennial root ornamental species (**Table 1**). Since adventitious bud regeneration is still the main regeneration pathway, the leaf is the most commonly used explant in ornamental plant transformation, while the protocorm is more used for bulbous species (Hoshi et al., 2004; Abbasi et al., 2020; Hirutani et al., 2020). Regeneration of woody species is still more challenging than that of other floral species; thus, the induction of somatic calli and the subsequent development of somatic embryos became an alternative method (Mishiba et al., 2005; Chin et al., 2007). Regardless of the regeneration system, the transformation efficiency and adaptability of cultivars vary among species, thereby impeding transgenic and gene editing research (Altpeter et al., 2016).

**Table 1:** *Agrobacterium*-mediated stable gene transformation in ornamental plants.

Species	Cultivar	Exogenous gene	Explant	Methods	*A. tumefaciens* strain	Transformation efficiency	Phenotype of transgenic plant	Ref.
*Rosa hybrida*	‘Samantha’	*GFP*	Leaf	Somatic embryogenesis	GV3101	5.0 ~ 6%	green fluorescence observed	(Liu G. et al., 2021)
*Rosa chinensis*	‘Old Blush’	*GUS*	Somatic embryos	Somatic embryogenesis+ Shoot regeneration	EHA105	ND	GUS positive	(Vergne et al., 2010)
*Eustoma grandiflorum*	‘Excalibur Pink’	*BEAT*	Flower	Floral-dipping	EHA105	1.5% (Pre-anthesis) 3.7% (Post-anthesis)	aromatic phenylacetate production	(Fang et al., 2018)
		*GFP*			/	/	In transgenic plants, green fluorescence can be clearly observed by stereo microscope.	
		AroG*			EHA105	1.1%	Not described.	
	X-1042	AroG*			EHA105	1.3%	Not described.	
	X-2541	AroG*			EHA105	0.2%	Not described.	
*Eustoma grandiflorum*	‘EX-Rosa Green’	*BAR*	Leaf	Shoot regeneration	LBA4404	0.6%	resistant to herbicides	(Chen et al., 2010)
*Tagetes erecta*	‘Xinghong’	*GFP*	Flower	Floral-dipping	EHA105	/	green fluorescence observed	(Cheng et al., 2019)
*Tagetes erecta*	line #39-7	*GUS*	Leaf	Shoot regeneration	LBA4404	/	Gus positive in leaves of transgenic plants	(Narushima et al., 2017)
*Chrysanthemum*	‘White Snowdon’	Artemisinin biosynthesis genes	Leaf	Shoot regeneration	CBE21	0.17%~0.33%	artemisinin production	(Firsov et al., 2020)
*Chrysanthemum*	‘Shinma’	*RsMYB1*	Leaf	Shoot regeneration	GV3101	1%	improved resistance to herbicides	(Naing et al., 2016)
					C58C1	3%	/	
					GV3101:C58C1=1:1	2%	/	
*Chrysanthemum*	‘Shuho-no-chikara’	*cry1Ab*	Leaf	Shoot regeneration	LBA4404	ND	Improved insect resistance	(Shinoyama et al., 2002)
*Campanula medium*	‘Blue double’	*GFP*	Leaf	Shoot regeneration	GV3101	0-12.7%	green fluorescence observed	(Gehl et al., 2020)
					AGL1	6.9-22.7%	/	
					ABI	0-7.6%	/	
*Campanula glomerata*	‘Acaulis’	*GUS*	Leaf	Shoot regeneration	EHA105	2%	Gus active	(Joung et al., 2001)
*Petunia hybrida*	‘Alvan’	*GUS*	Leaf	Shoot regeneration	LBA4404	0-22%	GUS positive	(Nobakht Vakili et al., 2018)
Lilium	‘Manissa’	*GUS*	Meristematic nodular calli	Shoot regeneration	EHA101	0-11.1%	stable expression of GUS gene	(Abbasi et al., 2020)
Lilium	‘Acapulco’	*GUS*	Filament-derived calli	Shoot regeneration	EHA101	/	GUS positive	(Hoshi et al., 2004)
*Begonia semperflorens*	/	*GUS*	Blisk	Tissue culture	EHA101	12-78%	GUS positive	(Hirutani et al., 2020)
*Dendrobium lasianthera*	/	*KNAT1*	Protocorm	Shoot induction	LBA4404	70%	KANT1 expression detected	(Utami et al., 2018)
*Cymbidium*	RY, L4, L23	*GUS*	Protocorm like body	Shoot induction	EHA101	/	GUS positive	(Chin et al., 2007)
*Phalaenopsis*	/	*GUS*	Protocorm	Shoot induction	EHA101	0.24~1.93%	GUS positive.	(Mishiba et al., 2005)|

Critical parameters for effective transformation include the species, cultivars, explant tissues, regenerative and transformation processes, induction medium, *Agrobacterium* strains, and phases. For instance, employing strain C58C1 resulted in a 3% transformation efficiency in the chrysanthemum cultivar ‘Shinma’, while using strain GV3101, a strain developed from C58C1, resulted in a 1% transformation efficiency (Gehl et al., 2020). Similarly, for the *Campanula medium*, the transformation efficiency was higher when using strain AGL1 as against GV3101 or ABI (Cheng et al., 2019). Floral dipping was developed for *Agrobacterium*-mediated genetic transformation to avoid the difficult and labor-intensive tissue culture (Clough and Bent, 1998). In ornamental plants, this method was successfully applied in *Eustoma grandiflorum* and *Tagetes erecta* (Fang et al., 2018; Cheng et al., 2019). These successful cases reveal the possibility to modify ornamental traits like scent in a simple and fast way, accelerating the breeding of ornamental plants, taking *Eustoma grandiflorum* for example (Fang et al., 2018). Even though it will take a lot of work to set up an effective system for crucial ornamental species, *Agrobacterium*-mediated gene transfer will serve as the backbone of transgenic breeding and genome editing.

## 3 Genome engineering: new strategies that revolutionize the future of ornamental breeding

Since model plants have revealed various unique technologies and methodologies in plant breeding, it is time to apply this knowledge to the breeding of ornamental plants. In order to provide innovative ways and tactics for ornamental plant breeding, this article focuses on the methodologies that have been extensively investigated and implemented in model plant breeding, from conventional cross-selection to molecular design breeding.

### 3.1 Manipulate recombination: the more genetic diversity, the more unpredictable traits

Over centuries, humans have used conventional crossbreeding procedures to develop an incredible range of phenotypic variability, from wildtype to commercial variants. The genetic variability of the offspring resulting from sexual reproduction is crucial to the success of selective breeding (Li F. et al., 2021). In sexual reproduction, meiosis reshuffles parental genomes through a specialized type of reductive cell division, which generates cells containing half of the chromosome complement with recombined parental genetic information (Li et al., 2017). It follows that the level of meiotic recombination is critically important for the resulting genetic diversity after breeding (Wijnker and de Jong, 2008). Consequently, plant breeders all over the world are working to increase recombination rates and the genetic diversity of their crops using a variety of methods, including the use of high-temperature (Francis et al., 2007; Wijnker and de Jong, 2008; Modliszewski et al., 2018) and radiation exposure (Raju and Lu, 1973; Singh, 1981; Jo et al., 2021), although the effect was not very significant. However, the main obstacle is that meiotic recombination is highly conserved and tightly regulated in plants, which leads to a low frequency of genetic exchange (Mercier et al., 2015). In addition, the distribution of meiotic recombination along chromosomes tends to cluster in a narrow region like telomere while rarely generated in the centromeric region (Wang and Copenhaver, 2018; Fernandes et al., 2019). As a result, in plant breeding, a high recombination frequency (RF) is highly prized since it reduces the impact of these factors on the genetic variants that arise during hybridization. Our understanding of the molecular mechanisms underlying meiotic recombination has greatly expanded thanks to recent advances in genetics, genomics, and bioinformatics, which in turn has helped speed up the plant breeding process and free genetic diversity from its inherent constraints (Davis et al., 2020; Kuo et al., 2021).

**Figure 1:**
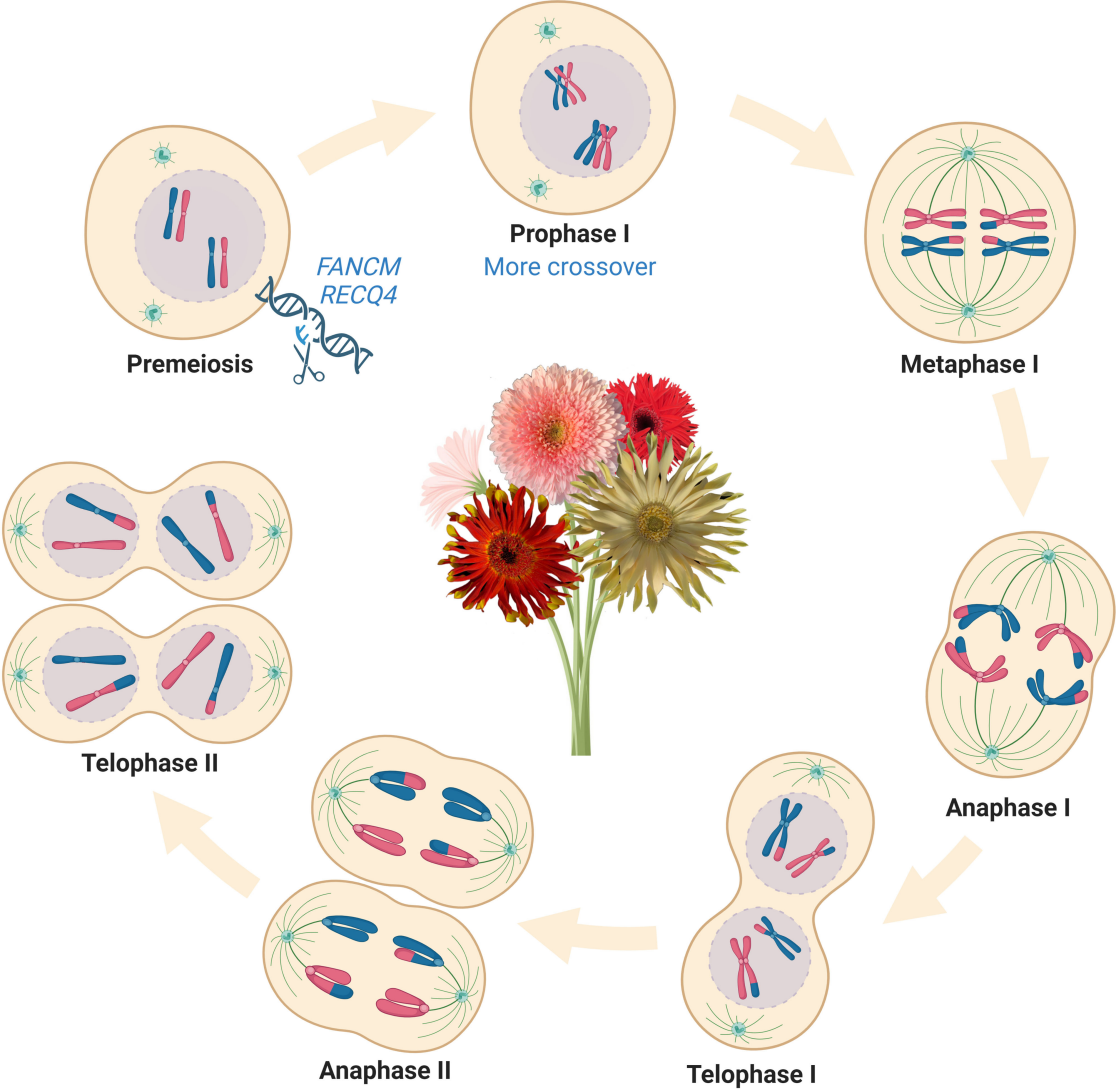
Manipulate meiotic recombination suppressors could boost massive crossover elevations in crop genomes and accelerate plant breeding programs. Knockout of *FANCM* and/or *RECQ4* can massively increase the crossover frequencies and genetic recombination, which provides a novel strategy for enriching genetic diversity in plant breeding. Abbreviations include FANCM, fanconi anemia complementation group M; RECQ4, recombination-deficient Q gene family. This figure was created with BioRender.com.

In the last decade, multiple anti-crossover (anti-CO) genes that limit meiotic recombination have been identified and their functions have been studied in *Arabidopsis*, including *FANCM (FANCONI ANEMIA COMPLEMENTATION GROUP M)*, *TOP3α (TOPOISOMERASE3α)*, *RECQ4 (RECQ HELICASE L4)*, *FIGL1 (FIDGETIN-LIKE-1)*, *HEI10 (HUMAN ENHANCER OF CELL INVASION NO.10)* and *HCR1 (HIGH CROSSOVER RATE1)* (Crismani et al., 2012; Séguéla-Arnaud et al., 2015; Hu et al., 2017; Séguéla-Arnaud et al., 2017; Ziolkowski et al., 2017; Fernandes et al., 2018a; Nageswaran et al., 2021). Knockout of single or multiple of these genes in *Arabidopsis* increases RF up to 10-fold, providing an emerging method to manipulate meiotic recombination in plants (Fernandes et al., 2018b). These tremendous advances in gaining insight into the genetic control mechanisms of meiotic recombination have made it possible to unlock genetic diversity that can be used for crop breeding, particularly with the help of the CRISPR genome editing system to precisely edit the genomes of higher plants and manipulate meiotic recombination. For instance, the RF of *fancm* mutants displayed a 3-fold increase in average compared with wildtype in *Brassicas* while displaying no defects in growth and fertility (Girard et al., 2014; Blary et al., 2018). The mutation of *RECQ4* can manipulate RF of different species, from a 6-fold increase in *Arabidopsis* to 3 folds in several other crops (rice, pea, tomato, and barley), suggesting that manipulating RECQ4 may be a versatile tool for boosting RF in plants (Mieulet et al., 2018; De Maagd et al., 2020; Arrieta et al., 2021). It needs to be noted that the distribution of CO events was not significantly changed in the *recq4* mutants, which limited its utility to elevate CO in the centromeric regions. In addition to anti-CO genes, CO interference, a phenomenon that one CO inhibits and prevents the formation of another one close to it in a distance-dependent manner along the chromosome also affects meiotic recombination (Sturtevant, 1915; Copenhaver et al., 2002; Berchowitz and Copenhaver, 2010, Otto and Payseur 2019). The natural variations in the meiotic crossover-promoting factor *HEI10* also contribute to regulating the crossover frequency and interference in a dosage-dependent manner, providing an alternative method to manipulate meiotic recombination in plants (Morgan et al., 2021). Overexpression of *HEI10* displayed a 2-fold increase in the genome-wide and showed a cumulative effect on crossover frequency (4-fold) when combined with the repression of *RECQ4* in hybrid plants (Serra et al., 2018). Understanding anti-CO genes and how they regulate meiotic recombination is a major step forward in plant breeding (**Figure 1**). Breeders are able to access previously untapped genetic diversity and generate novel combinations of desirable traits and unpredictable phenotypes with commercial value by directly introducing such hyper-recombinant traits into the genomes of superior crop cultivars.

In the field of ornamental plant research, due to the lack of efficient genome editing systems, whether these genes have the same function as crops needs to be further verified. Recently, six anti-CO factors were identified and studied in *G. hybrida* (Li F. et al., 2021; Li S. et al., 2021). These genes are highly expressed at the flower bud stage, but their expression levels drop dramatically later on, implying that they are involved in meiotic recombination in *G. hybrida*. This finding implies the potential for the application of anti-CO factors in ornamental breeding. However, one may concern about the impediments that limit the application. 1) the adaption of the gene functions. For example, the *fancm* mutants of *Arabidopsis*, as well as *Oryza sativa* and *Pisum sativum* exhibited RF elevation capacity, whereas no RF increase was detected in the *fancm* mutant of *Solanum lycopersicum* (Mieulet et al., 2018). Also, the polymorphism level in the hybrid context impairs the RF regulation efficiency in *Arabidopsis* hybrids (Girard et al., 2015; Ziolkowski et al., 2015) and the mutation of *FANCM* can only lead to the increase of RF when the polymorphism rate is lower than a certain threshold, such as 0.2 to 5 SNPs per kb (Zapata et al., 2016; Mieulet et al., 2018). 2) Defects caused by gene function loss. The *figl1* mutants in rice, pea, and tomato caused growth defects like impaired fertility (Zhang P. et al., 2017; Mieulet et al., 2018). Nevertheless, *RECQ4*, which overcomes the mentioned limits, is the most optimal candidate to be employed in plant breeding (Li F. et al., 2021), which provides the opportunity to create hyper-recombinant ornamentals using the anti-CO strategy.

### 3.2 Haploid breeding: one inducer serves all cultivars

The double haploid (DH) strategy is preferred by breeders due to its ability to fix the desired traits in an incredibly fast way. Nevertheless, spontaneous haploid production happens rarely in nature. Thus, many efforts have been made to induce haploid manually, including microspore/anther/ovule regeneration, interspecific cross, and haploid inducer (HI) (Dunwell, 2010; Chaikam et al., 2019).

**Figure 2:**
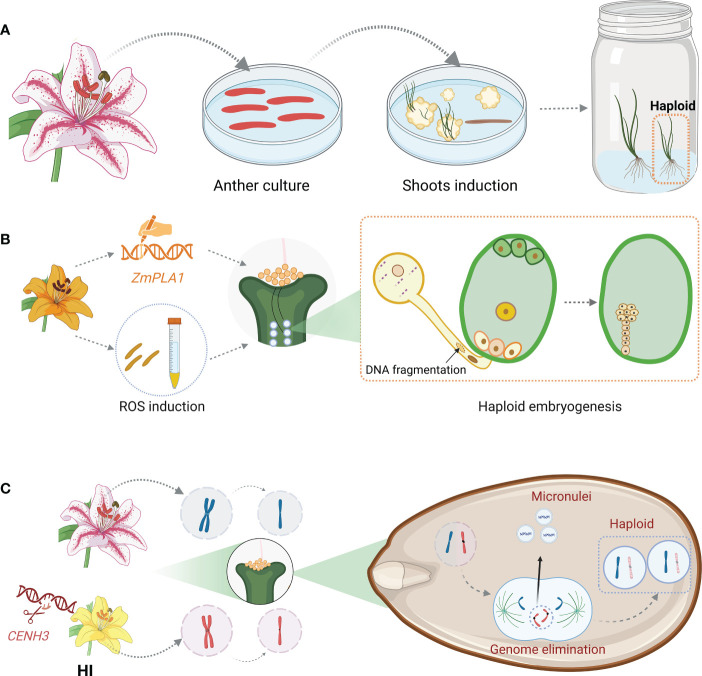
Summary of current haploid induction method in practice. **(A)** Haploid induction *via in vitro* culture. Anther, microspore, or unfertilized ovule are cultured for callus induction and adventitious shoots are regenerated from the calli, including haploid ones. **(B)** Haploid induction through pollen modification. Pollen either derived from a HI with mutated ZmPLA1 allele or pre-treated with chemicals to induce ROS are used for pollination. The modified pollen produces sperm with fragmented DNA which cannot be inherited after fertilization, leading to the production of haploid embryo. **(C)** Haploid induction based on CENH3 modification. HI is served as either female or male parent. After fertilization, the zygote enters embryogenesis procedure. The chromosomes inherited from HI parent lag during anaphase due to weak kinetochores. Those lagged chromosomes are eliminated from the main nuclei and form micronuclei, leading to the formation of haploid embryo. Abbreviations include ZmPLA1, Phospholipase A1; ROS, reactive oxygen species; CENH3, centromeric histone h3. This figure was created with BioRender.com.

The meagre reports on haploid induction in ornamental plants, including carnation, lily, marigold, chrysanthemum, and gerbera, were restricted in the *in vitro* techniques based on the regeneration of unfertilized ovule or microspores at early-uninucleate to the early-binucleate stage (Han et al., 1997; Sato et al., 2000; Wang et al., 2014; Kumar et al., 2019; Kumar et al., 2020; Li et al., 2020a) (**Figure 2A**). In the marigold case, 41.18% (7/17) regenerated plantlets were haploids when taking un-fertilized ovule as explant while 14.3% (8/56) dihaploids were regenerated from microspore culture. Obviously, the regeneration efficiency was higher in androgenesis whereas the subsequent haploid induction ratio (HIR) was lower compared with gynogenesis (Kumar et al., 2019; Kumar et al., 2020). In addition to the HIR, similar to wheat, various factors including bud stage, response to induction medium, cold pre-treatment, the period for dark culture, etc., affect the induction of haploid (Weigt et al., 2016; Kumar et al., 2019; Kumar et al., 2020). All of the mentioned factors have to be tested for the establishment of an applicable protocol. However, The enormous number of cultivars for each ornamental species makes it impossible to create haploids through this genotype-dependent method, which impedes the effectual application of the haploid induction strategy in ornamental breeding.

In commercial practice, mature haploid induction operation is achieved through HI-involved cross, taking maize as an example. The plant which can induce haploid progenies upon outcrossing either maternally or paternally, referred to as HI, was first reported in maize ‘Stock 6’ (Coe, 1959). Several QTLs associated with haploid induction capacity, including qhir1 and qhir8, were uncovered through fine mapping. These two QTLs contribute independently and HIs who own both QTLs display higher HIR than those owning a single QTL. For instance, the HIR of CAU5, a modern HI, which possesses both qhir1 and qhir8, is up to 10% (Liu et al., 2015). In the past decade, studies on ‘Stock 6’ deriving HIs demonstrated that NOT LIKE DAD (NLD)/MATRILINEAL (MTL)/PHOSPHOLIPASE A1 (ZmPLA1), a pollen-specific phosphatase, which localizes specifically on pollen cytoplasm was responsible for the loss of paternal genome during maize fertilization (Gilles et al., 2017; Kelliher et al., 2017; Liu et al., 2017). Nevertheless, the HIR varies in different situations. Deletions in *ZmPLA1* caused by CRISPR editing in the B73 background endowed the plants with an average haploid induction capacity at 2% (Liu et al., 2017), while the frameshift mutations in the *MTL* in the NP2222 background led to 6.67% haploid production (Kelliher et al., 2017). As *NLD*/*MTL*/*ZmPLA1* is conserved in monocots, its application is then extended to other cereals. Knockout of the orthologues of this gene led to 2-6% haploid progenies in rice (Yao et al., 2018) and 5.88-15.66% haploid offspring in wheat (Liu et al., 2020). It was hypothesized that sperm DNA fragmentation took place in the *nld*/*zmpla1*/*mtl* mutants, resulting in the uniparental genome elimination afterward. Further study on *zmpla1* mutant revealed that lipid imbalance was caused by impaired ZmPLA1 function, resulting in a ROS burst and DNA fragmentation in sperm. Interestingly, in addition to the important explanation of the haploid induction mechanism of *zmpla1*, this study demonstrated that ROS burst in plants either induced by chemical or by the mutation of peroxidase gene like *ZmPOD65*, endow the plant haploid induction capacity like a HI (Jiang et al., 2022) (**Figure 2B**).

Aside from *NLD*/*MTL*/*ZmPLA1*, the mutation of *ZmDMP*, a non-Stock 6-derived gene, existing in both monocots and dicots, also leads to the generation of maternal haploids (Zhong et al., 2019). It was then proved that without the function of the *ZmDMP*-like gene *AtDMP8* and *AtDMP9*, the *Arabidopsis* plant displayed a haploid induction capacity, with an average HIR of 2.2% (Zhong et al., 2020). Similarly, the *dmp8 dmp9* double mutants of *Medicago truncatula*, the model plant in legume, can produce 0.29-0.82% haploid progenies when self-pollinated and the HIR is 0.55% when it served as the pollen donor (Wang et al., 2021b).

Either the *NLD*/*MTL*/*ZmPLA1* or the *DMP* strategy hardly reaches the requirements of modern HI, which should be ~10% (Zhong et al., 2020), while another stronger toolbox based on CENH3 (CENTROMERIC HISTONE H3) modification was reported in 2010 by Ravi’s group (Ravi and Chan, 2010). The centromere is a critical region on a chromosome that directs chromatid segregation during mitosis and meiosis. It is composed of more than one hundred proteins, including CENH3, which are essential for kinetochore assembly (McKinley and Cheeseman, 2016). The knockdown of *CENH3* in *Arabidopsis* causes chromosome lagging and micronuclei formation during mitosis and meiosis (Lermontova et al., 2011), but rarely haploid induction upon the cross. By transferring the chimeric *AtCENH3* whose N-tail was replaced by *AtH3.3* into the *Arabidopsis cenh3* null mutant, the obtained tail-swap line (*GFP-tailswap*) could produce about 1/3 haploids when outcrossed as a female parent with wildtype and about 4% haploid progenies when serving as the pollen donor (Ravi and Chan, 2010). Alternative ways were provided to create *CENH3* modification-based HIs in *Arabidopsis*, including Site-Directed Mutagenesis, CRISPR, and EMS-mutagenesis, with the haploid induction rates varying from 2% to 44.1% (Karimi-Ashtiyani et al., 2015; Kuppu et al., 2015; Kuppu et al., 2020). Through CRISPR technology, this haploid induction strategy was also applied successfully in the cereals, with a haploid induction rate of ~7% in wheat (Lv J. et al., 2020) and ~5% in maize (Wang et al., 2021a).

It was believed that chromosomal instability brought on by poor CENH3 function leads to uniparental genome deletion during embryogenesis. Weak kinetochores are created when CENH3 is modified, and some of the chromosomes with these kinetochores trailed during anaphase. The lagging chromosomes were then expelled from the primary nucleus and developed into micronuclei. In addition to haploids, diploids, and aneuploids are produced as a result of the recruitment of some chromosomes into the main nucleus and the degradation of other chromosomes (Tan et al., 2015). Recent studies showed that direct degradation of CENH3 *via* a nanobody-based method produced haploids even though the prior study showed that RNAi-mediated suppression of CENH3 expression did not produce any haploids (Demidov et al., 2022) (**Figure 2C**).

Consequently, producing HIs would be the most practical method of inducing haploidy, and CENH3 is the best editing candidate. To date, the CENH3-based haploid induction system has been successfully established in *Arabidopsis*, wheat, maize, and switchgrass (Ravi and Chan, 2010; Karimi-Ashtiyani et al., 2015; Kuppu et al., 2015; Kuppu et al., 2020; Lv J. et al., 2020; Wang et al., 2021a; Yoon et al., 2022). As a particular histone 3, CENH3s in various species have a conserved histone folding domain (HFD) that is very similar to other H3 proteins but has a divergent N-terminal (Keçeli et al., 2020). It was reported that CRISPR system was able to modified all CENH3α A, B and D homoeologues in wheat by the same sgRNA targeting in the first and/or the intron 2/exon 3 splice site (N-terminal domain) and the resulting mutants produced up to 7% haploids upon out crossing (Lv J. et al., 2020). The ability to build CENH3-editing haploid induction systems in ornamental fields would be made possible by the acquisition of genomes and the development of CRISPR technology in an increasing number of ornamental species.

### 3.3 Maintain heterozygosity: clonal seed *vs* reverse breeding

Plants have undergone both natural and anthropogenic selection, resulting in a wide range of genetic variations and phenotypes. Heterosis refers to the phenomenon observed in modern breeding events in which offspring from a cross between two varieties within a species or between species have superior characteristics to those of either parent, including increased biomass, stronger resistance to biotic or abiotic stresses, and higher fertility (Wang et al., 2019). Most of the ornamental species popular in the market nowadays are hybrids while some of them are propagated by F1 seeds, such as garden petunia, marigold, and lisianthus. However, part of the traits is segregated while part of them tends to be homozygous upon selfing from generation to generation according to Mendel’s principle, accompanying the disappearance of the heterosis and the attractive characters. Thus, the elite F1 hybrids have to be created annually, which requires labor, field, and other producer goods. These efforts were reduced when the heterozygosity of the F1 hybrids was inherited through clonal seed (Wang et al., 2019).

**Figure 3:**
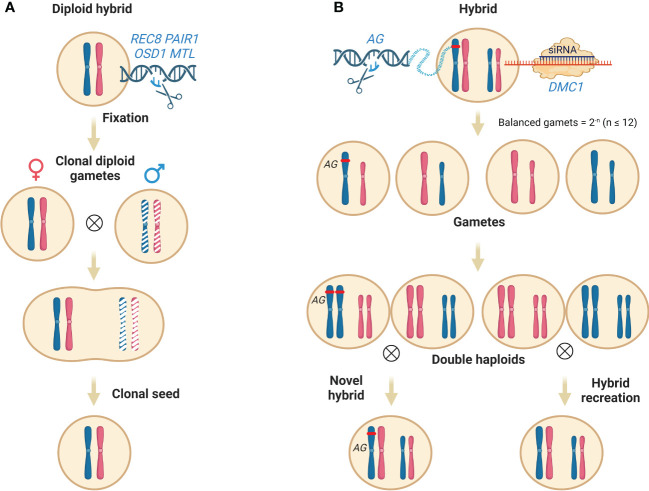
Maintain heterozygosity through clonal seed and/or reverse breeding. **(A)** Maintain heterosis of a tetra mutant. Firstly, the knockout of *REC8*, *PAIR1* and *OSD1* leads to the production of 2n pollen without meiotic recombination. Secondly, by introducing the mutation of haploid induction associated gene *MTL*, the extra paternal genome is eliminated upon fertilization, resulting in the production of clonal seeds. **(B)** recreation of hybrid by reverse breeding. Reverse breeding starts from a fully heterozygous hybrid by knock-down *DMC1* to suppress meiotic recombination, leading to the generation of balanced gametes without meiotic recombination at a certain percentage (2^-n^, n is the number of a set of chromosomes). A population of doubled haploids is then converted from the non-recombined haploids, which can be used to recreate hybrids. In addition, novel parental lines can also be created by genome editing of key genes that regulate desirable traits, such as the double-flower phenotype (*AG* for example). Abbreviations include REC8, meiotic recombination protein; PAIR1, homologous pairing aberration in rice meiosis1; OSD1, omission of second division; MTL, MATRILINEAL; AG, AGAMOUS; DMC1, disrupted meiotic cDNA1. This figure was created with BioRender.com.

The production of clonal seed contains two main steps: 1), the production of diploid (2n) pollen; 2) the elimination of the male or female parental genome. Plant male meiosis completes genome reduction through single DNA replication and two cycles of cell divisions (Hoshi et al., 2004). Defects in early meiotic events like chromosome cohesion, pairing and recombination, cell cycle progression, spindle organization, and cytokinesis would disrupt the regular path, leading to the formation of 2n pollen occasionally (Brownfield and Köhler, 2011). *AtSPO11-1 (* Grelon et al., 2001 *)*, *AtSPO11-2 (* Stacey et al., 2006 *)*, *PRD1 (* De Muyt et al., 2007 *)*, *PRD2 (* Walker et al., 2018 *)*, *PRD3*/*PAIR1 (* De Muyt et al., 2009 *)*, *DFO (* Zhang et al., 2012 *)*, *RAD51 (* Su et al., 2017 *)*, *DMC1* (Kurzbauer et al., 2012) and *MTOPVIB (* Tang et al., 2017 *)* are essential for meiotic recombination through DNA double-strand break formation, the function loss of which can abolish the meiotic recombination, resulting in no exchange of parental chromosome fragments. Next, the function loss of three kinds of genes can cause the production of 2n pollen formation. i) genes encoding cyclins essential for meiotic cell cycle progression whose absence leads to the omission of meiotic cell cycles, including *TARDY ASYNCHRONOUS MEIOSIS*/*CYCLIN-A 1;2* (*TAM/CYCA1;2*) and *OMISSION OF SECOND DIVISION* (*OSD1*) (d’Erfurth et al., 2010; Wang and Yang, 2014; Brownfield et al., 2015); ii) genes which are essential for proper formation and position of spindle, guaranteeing faithful chromosome segregation, like *JASON* and *AtPS1* (d’Erfurth et al., 2008; De Storme and Geelen, 2011); iii) genes essential for meiotic cytokinesis, including *ANP1* and *STUD*/*TES*/*AtNACK2* (Yang et al., 2003; Takahashi et al., 2010). Turning plant meiosis into mitosis (MiMe), scientists combined mutants involved in the above processes. The single mutant (*rec8*, *pair1*) or double mutant (*rec8 pair1*) which displays meiotic recombination defects cannot produce viable seeds whereas the triple mutant skips the second meiotic division (*rec8 pair1 osd1*) can (Mieulet et al., 2016). After the successful production of MiMe pollen, the scientist introduced the mutation of the genome elimination gene, *MTL* to eliminate the superfluous parental genome to keep the hybrids diploid. The subsequent quadruple mutant (*rec8 pair1 osd1 mtl*) can produce viable clonal seed (Wang et al., 2019). Moreover, except *REC8* and *PAIR1*, other genes mentioned above can be candidates to disrupt meiotic recombination, which extends the application of the MiME strategy in different crops (Mieulet et al., 2016) (**Figure 3A**).

In addition to the cloning of F1 hybrids, regaining and extension of parental resources are also critical for cultivar innovation. Reverse breeding is an unprecedented approach that meets the challenge by suppressing meiotic recombination to generate perfectly complementing homozygous parental lines from the heterozygous plants (Dirks et al., 2009) (**Figure 3B**). Wijnker et al. proved the feasibility of this method in *Arabidopsis via DMC1* silencing (Wijnker et al., 2012). DMC1 is a meiosis-specific recombinase essential for the formation of crossover and recombination (Hinch et al., 2020). Knock-down of DMC1 caused random segregation of the non-recombinant chromosomes during meiosis, leading to unbalanced chromosome segregation and aneuploid gamete formation (Couteau et al., 1999) whereas viable balanced gametes without CO were produced occasionally at a certain frequency in *Arabidopsis* (2n = 5) at a theoretical frequency of 3.25% (2^-5^). These viable gametes harboring non-recombinant parental chromosomes can be cultured *in vitro* to regenerate haploid plantlets then (Wijnker et al., 2014). As RNA interference functions in the transgenic hybrids, non-transgenic plantlets can be found in the regenerated shoots which display normal fertility after chromosome doubling. In contrast, the complete knockout of *DMC1* will reintroduce the mutation into the offspring and impair their fertility.

Along with the haploid gametes regeneration, the haploid homozygous plant can also be accessed through the haploid induction cross mentioned above where the *DMC1* silenced hybrid, referred to as reverse breeding F1 served as the pollen donor. Upon haploid induction crosses, the maternal genome of HI is eliminated while the paternal F1 genome without recombination can be inherited by chance. DH can be achieved by occasionally happened natural chromosome doubling or by chemical treatment. Interestingly, since the uniparental genome is inherited specifically, we can create novel parental lines by introducing genome editing during reverse breeding. For example, when an F1 hybrid which is *DCM1* silencing and *AG* (*AGAMOUS*) knock-out is used for reverse breeding, the outcome of a parental plant possessing an extra double-flower trait is liable.

## 4 Conclusions and perspectives

Over the centuries, plant breeders have produced a wide variety of cultivars with rich traits, and the regulation mechanisms of some have been revealed in past decades with the development of molecular biology. The progressive accumulation and validation of knowledge valuable for plant breeding in model plants such as *Arabidopsis* and rice have established a theoretical foundation for ornamental plant breeding. Recent advances in genomics have significantly advanced basic research in horticulture plants, particularly with the successful application of genome editing technologies, implying a new avenue for ornamental research and breeding. However, additional research is required due to the complexity of the genetic background and breeding history of ornamentals.

Despite the breakthrough in technological advances of model plant genome editing, its application in ornamentals remains immature and inefficient. As of 2022, assembled genome sequences of more than 100 ornamental plants have been released, whereas the number of species possessing a genome editing system is less than 20 (Zheng et al., 2021; Duan et al., 2022). It’s possible that a combination of factors, including a deficiency in effective and genotype-independent genetic transformation processes, is responsible for this observation. For example, despite years of effort, the stable *Agrobacterium*-mediated genetic transformation protocol for *Gerbera hybrida* was only developed for an old cultivar called ‘Terra Regina’ (Elomaa and Zhang, 2022). This is a widespread problem reported in most ornamental plants, including roses, carnations, and eustoma. Recent new findings have revealed that altering genes in the *WOX* family can disrupt genetic requirements for plant regeneration and transformation, offering an opportunity to develop a more efficient transgenic pathway for ornamental plants (Wang et al., 2022). Development regulators involved in plant regeneration are continuously being uncovered. Transcription factors like *SERK1/2*, *PLT3/5/7*, *ABI3*, etc. are included and the molecular network regulating *novo* shoot regeneration are being drawn and extended (Sugimoto et al., 2019). The second barrier is the low efficiency of genome editing, which may be associated with the adaption of the CAS system when transferring from model plant to ornamental plant, and the complex genetic background of the targets (highly heterozygous, polyploidization, etc.). To make ornamental breeding the new track, researchers must overcome this formidable obstacle. New opportunities for ornamental plant breeding will be opened up through a deeper understanding of molecular biology and genome engineering. More effective, systematic and targeted breeding strategies will revolutionize the future of ornamental horticulture and boost a greater variety of ornamental traits.

## Author contributions

CJ and FL wrote the manuscript. LD and CW collected the data. MW polished the language and revised the manuscript. CY participated in the discussion of the possibility of strategies in ornamental plant breeding. SL and FL critically revised the manuscript and provided supervision. All authors contributed to the article and approved the submitted version.
